# Immune-Mediated Inflammatory Diseases Awareness and Management among Physicians Treating Patients with Inflammatory Bowel Disease: An IG-IBD Survey

**DOI:** 10.3390/jcm13071857

**Published:** 2024-03-23

**Authors:** Marta Vernero, Cristina Bezzio, Davide G. Ribaldone, Flavio A. Caprioli, Massimo C. Fantini, Stefano Festa, Fabio S. Macaluso, Ambrogio Orlando, Daniela Pugliese, Sara Renna, Antonio Rispo, Edoardo V. Savarino, Angela Variola, Simone Saibeni

**Affiliations:** 1 Department of Medical Sciences, University of Turin, 10126 Turin, Italy; marta.vernero@unito.it (M.V.); davidegiuseppe.ribaldone@unito.it (D.G.R.); 2 IBD Centre, IRCCS Humanitas Research Hospital, 20089 Rozzano, Italy; cristina.bezzio@hunimed.eu; 3 Department of Biomedical Sciences, Humanitas University, 20072 Pieve Emanuele, Italy; 4 Gastroenterology and Endoscopy Unit, Fondazione IRCCS Ca’ Granda Ospedale Maggiore Policlinico, 20122 Milan, Italy; flavio.caprioli@gmail.com; 5 Department of Pathophysiology and Transplantation, Università degli Studi di Milano, 20122 Milan, Italy; 6 Department of Medical Science and Public Health, University of Cagliari, 09042 Cagliari, Italy; massimoc.fantini@unica.it; 7 Gastroenterology Unit, Cittadella Universitaria di Monserrato, 09042 Cagliari, Italy; 8 IBD Unit, San Filippo Neri Hospital, 00135 Rome, Italy; festa.stefano@gmail.com (S.F.); sararenna.md@gmail.com (S.R.); 9 IBD Unit, Villa Sofia Cervello Hospital, 90146 Palermo, Italy; fsmacaluso@gmail.com (F.S.M.); ambrogiorlando@gmail.com (A.O.); 10 CEMAD—IBD Unit, Department of Medical and Surgical Sciences, Fondazione Policlinico Universitario “A. Gemelli” IRCCS, 00168 Rome, Italy; daniela.pugliese@policlinicogemelli.it; 11 Department of Clinical Medicine and Surgery, “Federico II” University of Naples, 80131 Naples, Italy; antonio.rispo2@unina.it; 12 Department of Surgery, Oncology and Gastroenterology, University Hospital of Padua, 35128 Padua, Italy; edoardo.savarino@gmail.com; 13 IBD Unit, IRCCS Sacro Cuore Don Calabria, 37024 Negrar di Valpolicella, Italy; angela.variola@gmail.com; 14 IBD Centre, Gastroenterology Unit, Rho Hospital, ASST Rhodense, 20017 Rho, Italy

**Keywords:** IMID, IBD, Crohn’s disease, ulcerative colitis, healthcare, survey, multidisciplinary approach

## Abstract

(1) **Background**: Inflammatory bowel disease (IBD) is frequently associated to other immune-mediated inflammatory diseases (IMIDs). This study aims at assessing physicians’ awareness of the issue and the current status of IMID management. (2) **Methods**: A web-based survey was distributed to all 567 physicians affiliated to IG-IBD. (3) **Results**: A total of 249 (43.9%) physicians completed the survey. Over 90% of the responding physicians were gastroenterology specialists, primarily working in public hospitals. About 51.0% of the physicians had access to an integrated outpatient clinic, where gastroenterologists collaborated with rheumatologists and 28.5% with dermatologists. However, for 36.5% of physicians, integrated ambulatory care was not feasible. Designated appointment slots for rheumatologists and dermatologists were accessible to 72.2% and 58.2% of physicians, respectively, while 20.1% had no access to designated slots. About 5.2% of physicians report investigating signs or symptoms of IMIDs only during the initial patient assessment. However, 87.9% inquired about the presence of concomitant IMIDs at the initial assessment and actively investigated any signs or symptoms during subsequent clinical examination. (4) **Conclusions**: While Italian physicians recognize the importance of IMIDs associated with IBD, organizational challenges impede the attainment of optimal multidisciplinary collaboration. Efforts should be directed toward enhancing practical frameworks to improve the overall management of these complex conditions.

## 1. Introduction

Inflammatory bowel disease (IBD) is an immune-mediated disease involving the gastrointestinal tract. The primary forms of IBD are ulcerative colitis (UC) and Crohn’s disease (CD), which differ mainly in terms of intestinal location and depth of inflammation within the intestinal wall [1,2]. 

IBD should be recognized as a systemic disease since it typically extends beyond the gut. A significant number of patients with IBD have at least one other concomitant immune-mediated inflammatory disease (IMID) and/or exhibit extra-intestinal manifestations [3,4]. The difference between extra-intestinal manifestation of IBD and associated IMIDs can sometimes be debated. Indeed, several classical extra-intestinal manifestations, such as primary sclerosing cholangitis, axial or peripheral spondyloarthropathy, erythema nodosum, pyoderma gangrenosum, and uveitis, are de facto IMIDs. 

Furthermore, it is well established that several other IMIDs may be associated to IBD. For instance, among the most frequently associated IMIDs, numerous dermatological inflammatory conditions, such as psoriasis and atopic dermatitis [5], as well as rheumatological diseases, such as rheumatoid arthritis [6], are among the most common. Moreover, numerous rare IMIDs occasionally associate to IBD, including vitiligo, epidermolysis bullosa, polyarteritis nodosa, primary biliary cholangitis, granulomatous hepatitis, autoimmune pancreatitis, type 1 diabetes mellitus, autoimmune thyroid diseases, multiple sclerosis, and asthma [4]. 

Additionally, numerous IMIDs may paradoxically be induced by IBD therapies. For instance, many cases of new onset psoriasis during anti-TNF therapy for IBD have been reported [7]. Moreover, erythematous systemic lupus [8] and demyelinating diseases may occur in patients with IBD undergoing anti-TNF therapies [9]. Conversely, certain treatments used for rheumatological and dermatological IMIDs, such as the anti-IL 17 monoclonal antibody secukinumab, may induce de novo IBD or worsen the clinical course of a concomitant IBD. 

The progressive worsening course of IMIDs, the necessity for advanced and potentially harmful therapies, and their resulting psychological and physical toll contribute to their status as a significant healthcare challenge, further compounded by a substantial economic burden. Moreover, it has been observed that patients with IMIDs have an increased risk for other comorbidities, including infections, renal and cardiovascular diseases, neoplasms, and psychiatric disorders [10,11,12]. Additionally, the presence of co-occurring IMIDs has been shown to negatively affect the course of IBD [13]. Consequently, the overall management of patients with IBD and concomitant IMIDs is more complex, and clinical decision-making becomes more challenging. Therefore, a multidisciplinary approach is preferable when a patient suffers from more than one IMID. Lately, there has been a growing recognition of the necessity for a new collaboration with nutritionists. A recent review showed that patients affected by different IMIDs tend to adopt different types of self-imposed restrictive diets, often due to insufficient nutritional care from their physicians [14]. 

It is advantageous for diverse specialists to collaborate in providing optimal treatment for all IMID manifestations, rather than addressing each IMID individually. Therefore, when managing patients with IBD, physicians should investigate the presence of coexisting IMIDs [15]. For instance, several indicators, referred to as “red flags”, have been suggested for the diagnosis of spondyloarthropathies [16,17]. Di Carlo et al. [16] proposed the DETAIL questionnaire, which consists of 6 items that assess joint involvement, such as low back pain at rest that improves with movement and a history of swollen fingers or joints. Similarly, also Ribaldone et al. [17] validated these items (via a modified STRII questionnaire) for identifying patients who require rheumatological evaluation. Conversely, certain major and minor “red flags” for IBD have been proposed as a tool for early referral to gastroenterologists [18]. 

The increasing recognition and recommendation of a multidisciplinary approach for patients with IBD are widespread, yet it remains uncertain whether physicians treating IBD consistently address the presence of concomitant IMIDs and have the practical possibility to collaborate with other specialists in managing IMIDs associated to IBD. 

The objective of our study is to evaluate Italian physicians’ awareness regarding these issues and to explore the availability of a shared pathway for the management of multiple IMIDs. 

## 2. Materials and Methods

### 2.1. Type of Study

This paper presents the results of a survey conducted among a large cohort of Italian physicians involved in the management of IBD. 

### 2.2. The Survey

A panel of experts from the Italian Group for the Study of Inflammatory Bowel Disease (IG-IBD) developed a questionnaire to explore the multidisciplinary assessment and management of patients with IBD, specifically focusing on the coexistence of IMIDs. The questionnaire was evaluated, revised, and endorsed by the Scientific Committee of IG-IBD. The final version included 20 questions (allowing single or multiple answers) and is provided as Supplementary Materials. Eight items of the questionnaire included general information about physicians and their work environment, namely sex, age, geographic location of the hospital, specialization, type of hospital, years of IBD clinical activity, age of patients, and the number of patients under follow-up in the center. Five items investigated the availability of various specialists in the hospital and the methods employed to ensure their evaluation for patients with IBD (such as shared outpatient visit, reserved slots, availability for on-demand evaluation, and remote on-demand consultations). Five additional items assessed physicians’ feelings and attitudes regarding the associations between IBD and concomitant IMIDs. Finally, one question regarded identifying the most relevant extra-intestinal specialist in the management of patients with IBD, while another question (rated on a scale from 0 to 10) addressed the relevance of IMID awareness in the management of patients with IBD. 

A formal invitation to participate in the survey was distributed via e-mail to all the regularly registered members of IG-IBD, in order with the annual membership fee. The questionnaire was implemented in a dedicated website. Physicians completed it between January and June 2023. No formal exclusion criteria were applied. 

### 2.3. Statistical Analysis

The data were extracted on an Excel^®^ 2010 datasheet and analyzed using MedCalc^®^ software, Version 5, Ostend, Belgium. For categorical variables, frequency was expressed in absolute numbers and relative percentages, while for continuous variables, the mean with the corresponding confidence interval was calculated for normally distributed variables, and the median and interquartile range (IQR) were calculated for non-normally distributed ones. Normal distribution was checked with the D’Agostino Pearson test. 

The impact of the variable of interest (sex, age, hospital geographic location, type of hospital, patient load, and specialization) on outcomes related to physicians’ awareness on IMIDs (including the estimated percentage of IBD patients with concomitant IMID, the given importance to IMID from 1 to 10, and the frequency of assessment of other IMIDs) was analyzed through logistic regression analysis. All variables reaching statistical significance at the univariate analysis were included in the multivariate analysis. When necessary, confidence intervals were computed at 95%, and the significance level was set for a *p*-value lower than 0.05. 

## 3. Results

### 3.1. Characteristics of the Responding Physicians

Out of 567 physicians, 249 (43.9%) completed the survey. 

Table 1 summarizes the general characteristics of the included physicians. Notably, the majority of the physicians (41.0%) were aged over 45 years and practiced in northern Italy (47.4%). Additionally, the vast majority of responding physicians were gastroenterologists (90.4%).

### 3.2. Characteristics of Working Places of the Responding Physicians

The features of the working places of the responding physicians are shown in Table 2. A majority were exclusively managing adult patients with IBD (71.9%), and 40.2% of them were following more than 1000 patients with IBD at their Center. 

### 3.3. Presence of Specialists Potentially Involved in Management of IMIDs Associated with IBD and Modalities of Consultation

**Figure 1:**
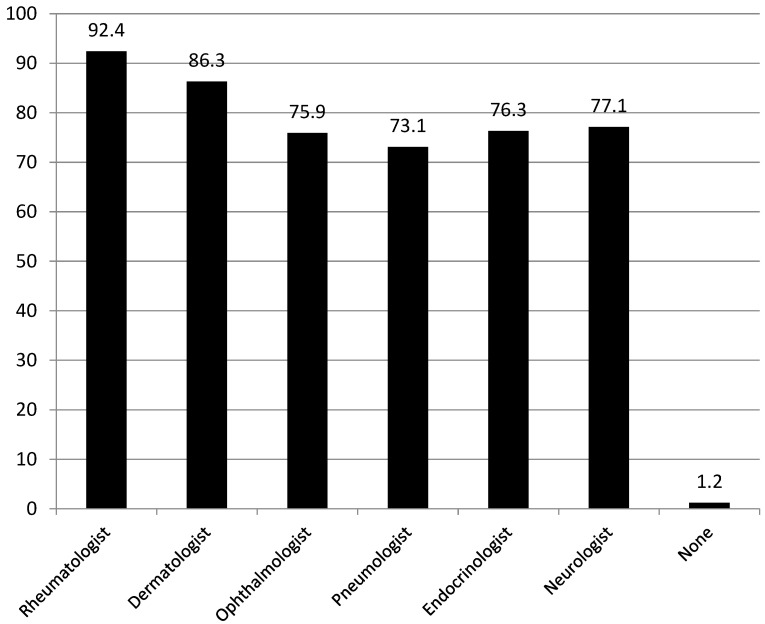
Presence of the different specialists involved in the management of IMIDs associated with IBD (expressed as percentages).

The presence of the different specialists involved in the management of IMIDs associated with IBD is expressed in Figure 1. 

High percentages of the responding physicians reported the presence in the same hospital of a rheumatologist (92.4%), a dermatologist (86.3%), and an ophthalmologist (75.9%). Notably, 3 physicians do not have any specialist available in their working hospital. 

Table 3 presents the methods of collaboration with various specialists involved in managing IMIDs. A shared ambulatory denotes a scheduled visit in a shared outpatient clinic setting where both specialists are simultaneously present. Alternative options included the availability of reserved slots for patients with IBD in other specialists’ ambulatory, the possibility to schedule on-demand visits, or remote consultation of cases. 

### 3.4. Attitudes and Feelings of Physicians Managing Patients with IBD toward IMIDs

We inquired about the perceived percentage of patients with IBD with concomitant IMIDs among physicians: 37 out of 249 (14.9%) responders believe that fewer than 10% of patients have a concomitant IMID, 138 (55.4%) estimate that 10 to 20% of patients with IBD have a concomitant IMID, and 72 (28.9%) estimate this percentage between 20 and 50%. None of them believe that more than 50% of patients have a concomitant IBD. 

A total of 219 (87.9%) physicians inquire for the presence of concomitant IMIDs at initial evaluation and actively check for signs or symptoms during subsequent clinical evaluations. Out of 249 physicians, 13 (5.2%) stated that they only investigate signs or symptoms of IMIDs only at the initial evaluation, 15 (6.0%) do so only if patients report a past history of IMID, and 2 (0.8%) indicated that they do not conduct such evaluations.

Physicians were also asked to rate the importance of concomitant IMID in IBD management on a scale from 1 to 10, and the median value of their answers was 10 (IQR 8–10).

**Figure 2:**
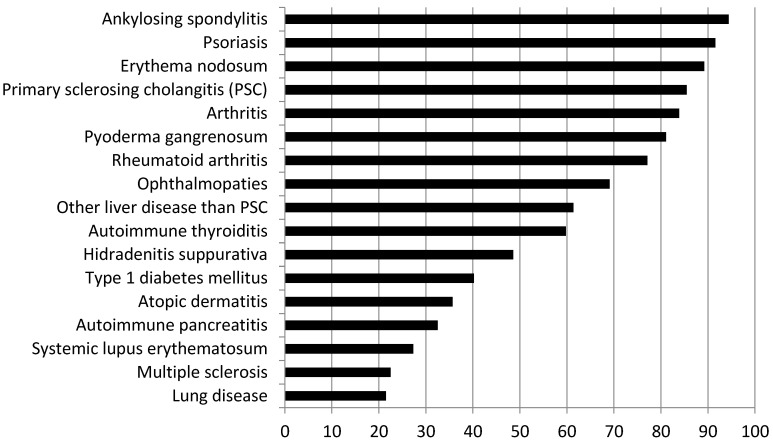
IMIDs more frequently taken into account during patients’ evaluation (expressed as percentages).

Figure 2 shows the IMIDs predominantly considered by physicians during routine evaluations. Overall, as depicted in the chart, rheumatologic, including axial and peripheral spondyloarthropathies, and dermatological diseases, such as erythema nodosum, psoriasis, and pyoderma gangrenosum, are the most frequently investigated, along with primary sclerosing cholangitis. On the contrary, systemic lupus erythematosus, multiple sclerosis, and lung disease are the least frequently considered IMIDs by responding physicians. 

Regarding the impact of IMIDs on IBD management and clinical course, 232 out of 249 (93.2%) of the interviewed physicians answered that the presence of concomitant IMIDs can impact therapeutic choices, such as opting for treatments with an overall effectiveness on coexisting conditions. According to 136 out of 249 (54.6%) physicians, certain drugs used for IBD may be contraindicated for specific IMIDs, while treatments for certain IMIDs can potentially worsen the progression of IBD. A total of 121 (41.0%) physicians assert that the presence of concomitant IMIDs per se may worsen the course of IBD, while 5 (2.0%) suggest that coexisting IMIDs may have a positive influence on the course of IBD. One physician stated that the presence of IMIDs influences neither the management of IBD nor its course. 

Based on our survey, 151 out of 249 (60.6%) physicians have access to a multidisciplinary team comprising different specialists collaborating to improve the management of the IMIDs. Ninety-four (37.7%) physicians manage IBD themselves and delegate the management of other IMIDs to designated specialists. Furthermore, 4 (1.6%) physicians independently handle both IBD and all other IMIDs. 

Lastly, we requested physicians to identify a specialist whom they deem essential when dealing with patients with both IBD and other IMIDs. The vast majority (213/249) indicated a preference for rheumatologists while 16 (6.4%) for dermatologists, 3 for neurologists, 2 for endocrinologists, 1 for ophthalmologists, and no one for pneumologists. A total of 14 (5.6%) indicated that no specialist is essential for managing IMIDs associated with IBD. 

### 3.5. Factors Influencing Physicians’ Answers

Table 4 presents the findings from univariate analysis regarding factors that may influence the importance attributed by physicians to IMIDs, the estimated percentage of IMIDs within IBD population, and the frequency of assessing the presence of concomitant IMIDs.

We considered age (more than 45 years), sex (male), location of the hospital (northern Italy), specialization (gastroenterology), type of hospital (academic), and patient load (more than 1000).

Regarding the significance attributed by physicians to concomitant IMIDs, none of the analyzed variables showed a significant correlation with the outcome. Consequently, we did not conduct multivariate analysis for this aspect. 

Concerning the estimated percentage of patients with IBD with a concomitant IMID (>20%), only the patient load exceeding 1000 demonstrated a significant association with the outcome (OR 3.40; 95% CI 1.42–8.10; *p* = 0.005). As the patient load was the only significant variable in the univariate analysis, we included the university hospital variable in the multivariate analysis as it exhibited the closest result to significance in the univariate analysis (OR 1.89; 95% CI 0.90–3.9; *p* = 0.09) and as it was considered potentially important for the outcome. In the multivariate analysis, only patient load over 1000 of patients confirmed its significant influence on the outcome (OR 1.54; 95% CI 1.16–3.20; *p* = 0.01).

In the univariate analysis, the probability of assessing IMIDs at each visit was significantly higher for physicians working in university hospitals (OR 2.48; 95% CI 1.12–5.81; *p* = 0.03). In the multivariate analysis, working in a university hospital was confirmed to increase the likelihood of actively seeking associated IMIDs at each visit (OR 2.40; 95% CI 1.10–5.70; *p* = 0.04). 

## 4. Discussion

In this study, via the use of a web-based questionnaire, we assessed the awareness among physicians managing patients with IBD regarding the presence of concurrent IMIDs, along with their attitudes in this peculiar clinical setting. 

To the best of our knowledge, this field has been somewhat overlooked until now, and we believe that our findings provide valuable insights for enhancing the management of these conditions.

The percentage of responders (around 45%) is even higher than that reported in previous surveys of similar nature [19,20,21].

As expected, the vast majority of responding physicians was represented by gastroenterologists. The majority of respondents were based in northern Italy, mostly in public hospitals, where they treated adult patients with IBD, managing a patient load higher than 500 individuals and possessing over 15 years of experience in managing patients with IBD. The age of distribution among the respondents was fairly balanced. 

Overall, Italian physicians seem to be well aware about the potential presence of a concomitant IMID in their patients with IBD and recognize the importance of investigating this association. Indeed, the median answer to the question “how much do you think this association between IBD and IMID is important in IBD management (from 1 to 10)” was 10 (IQR 8–10), and the majority of responding physicians correctly estimated the prevalence of their patients with IBD with at least one other concomitant IMID. Indeed, as indicated in the literature, this prevalence typically ranges from 20 to 25% [22,23]. Moreover, they possess a clear understanding that the concomitant presence of IMIDs implies integrated and challenging therapeutic decisions as well as mutual influences on the progression of each disease. These observations are consistent with findings provided by literature [9,10,11,12,13].

From a practical standpoint, over 90% of facilities have access to at least one rheumatologist, and nearly 90% have access to at least one dermatologist. Additionally, approximately 75% of physicians have access to an ophthalmologist, an endocrinologist, or a neurologist within their working hospitals. 

Regarding collaboration practices, half of the respondents have the opportunity to a shared ambulatory with rheumatologists and 1 out of 3 with dermatologists. Concerning the availability of reserved slots for patients with IBD in various outpatient clinics, the majority of physicians have this option with rheumatologists and dermatologists. Furthermore, for on-demand evaluation, opportunities are definitively more extensive, with nearly 85% of respondents having this possibility with rheumatologists and around 75% with dermatologists. Additionally, nearly half of them have similar opportunities with endocrinologists, neurologists, and pneumologists. On the contrary, almost 40% of physicians do not have any shared outpatient clinic, and 1 out of 5 do not have the possibility of reserved slots or remote consultations. 

In terms of IMIDs of major interest, rheumatologic diseases receive the greatest focus, particularly with emphasis on conditions such as ankylosing spondylitis, rheumatoid arthritis, and other forms of arthritis, including psoriatic arthritis and juvenile idiopathic arthritis. Indeed, spondyloarthropathies and psoriatic arthritis have been reported to be the most common co-occurring rheumatologic disease in patients with IBD [24]. 

In addition to the typical cutaneous manifestations of IBD, such as erythema nodosum and pyoderma gangrenosum, which are routinely assessed by most physicians treating patients with IBD, hidradenitis suppurativa and atopic dermatitis are dermatological IMIDs of major interest for specialists in IBD. Of note, hidradenitis suppurativa is diagnosed in approximately 12% of IBD population, indicating a higher prevalence compared with the general population (nearly 1%) [25]. Furthermore, a two-way association between atopic dermatitis and IBD has been suggested [26]. 

When questioned about the indispensable specialist in managing IBD and IMIDs, the vast majority of the interviewed physicians identified rheumatologists as essential. Indeed, the collaboration between gastroenterologists and rheumatologists appears to be quite satisfactory in Italy. This collaborative effort has already provided guidelines to improve the referral and management of patients with concomitant IBD and spondyloarthropathies [16,17,18]. 

Based on our survey findings, academic hospitals and physicians managing a high patient load demonstrate greater awareness regarding possible association with IMIDs. Furthermore, IBD specialists working in academic hospitals are more inclined to actively seek signs of associated IMIDs during scheduled visits. One possible explanation could be that institutions with academic affiliations and those managing larger patient volume are more inclined and better positioned to assess the association between IBD and IMIDs as well as to undertake clinical studies investigating different concomitant IMIDs. Despite the higher representation of responding physicians from northern Italy, no significant associations were found with geographic location. 

This survey presents the current perspective of Italian physicians who manage patients with IBD regarding the presence of concomitant IMIDs, providing an overview of their typical approach to managing patients with IBD in this peculiar setting. 

Regarding the negative findings, it is notable that not all the centers have the possibility to establish a shared ambulatory with rheumatologists and/or dermatologists, which is crucial when IBD is associated with rheumatologic or dermatological IMIDs to ensure optimal overall management. Indeed, a joint evaluation involving both gastroenterologists and dermatologists or rheumatologists is essential when considering target therapy prescription. Some treatments may be indicated for both the diseases, while others may be ineffective or even contraindicated for one of the IMIDs. For instance, anti-IL17 drugs are highly effective in treating psoriatic arthritis [27], but they are contraindicated in IBD [28]. Conversely, anti-integrin therapies like vedolizumab are effective in treating IBD [29] but do not affect the joints or the skin. 

We consider the results of our survey to be factual, interesting, innovative, and reliable. These findings are derived from physicians affiliated to the Italian Group of Inflammatory Bowel Disease, exhibiting an equal distribution across demographic, clinical, and structural features. Possible limitations of our survey may include inherent aspects typical of this type of study, such as the utilization of sampled (rather than complete) data, the subjective nature of responses, the presence of unintentional biases, the absence of validation of the questionnaire, and the impossibility to decide the exact number of participants. Another limitation may be represented by the fact that, although the sample seems to be adequately representative of the situation in Italy, our observations cannot universally be generalized. In this regard, it would be interesting to conduct studies directly comparing physicians from different countries to assess similarities and differences about this emerging topic. Finally, as this survey was administrated to physicians referring to IG-IBD, it may not fully represent the entire population of gastroenterologists, since they constitute a selected population of clinicians accustomed to managing IBD in specialized settings. 

The concomitant presence of IMIDs in a single patient is becoming increasingly interesting to physicians, as evidenced by the even more emerging and more precisely defined concept of different-targeted therapies [30]. However, in the multifaceted real-world setting, it is possible that physicians may not always possess the requisite knowledge and/or the resources to effectively manage these conditions. In this regard, we believe that the results of this survey serve as a crucial starting point for raising awareness regarding the presence of concomitant IMIDs in patients with IBD. 

## 5. Conclusions

Italian physicians managing patients with IBD recognize the importance of the association between IBD and various IMIDs. However, practical arrangements may not always support them in their daily clinical practice. Further efforts are needed to facilitate a multidisciplinary approach, ultimately improving the management of these conditions. One possibility may involve the active collaboration among various scientific societies to raise awareness about the presence of concomitant IMIDs within their respective specialist milieus, thereby enhancing the overall management, including clinical outcomes, for affected patients. Moreover, the identification of “red flags”, similar to those developed between gastroenterologists and rheumatologists, would lead to appropriate mutual referrals, ensuring early and correct diagnosis crucial for reducing the progressive detrimental course of individual IMIDs. 

## Figures and Tables

**Table 1:** Characteristics of the responding physicians.

		**n (%)**
**SEX**	*Men*	135 (54.3)
	*Women*	114 (45.7)
**AGE**	*25–34 years old*	73 (29.3)
	*35–44 years old*	74 (29.7)
	*>45 years old*	102 (41.0)
**PART OF ITALY**	*North*	118 (47.4)
	*Center*	79 (31.7)
	*South*	51 (20.5)
**TIME IN IBD FIELD**	*<5 years*	70 (28.1)
	*5–15 years*	77 (30.9)
	*>15 years*	102 (41.0)
**SPECIALIZATION**	*Gastroenterology*	225 (90.4)
	*Internal medicine*	17 (6.8)
	*Pediatrics*	4 (16)
	*Surgery*	1 (0.4)
	*Pathologist*	1 (0.4)

**Table 2:** Characteristics of the working environment.

		**n (%)**
**Working hospital**	*Public community*	123 (49.4)
	*Public academic*	64 (25.7)
	*Private community*	10 (4.0)
	*Private academic*	22 (8.8)
	*Private practice*	4 (1.6)
	*Not reported*	26 (10.5)
**Age of patients**	*>18 years*	179 (71.9)
	*14–18 years*	64 (25.7)
	*<14*	6 (2.4)
**Patient load**	*<500 patients*	62 (24.9)
	*500–1000*	87 (34.9%)
	*1000–3000*	64 (25.7%)
	*>3000*	36 (14.5%)

**Table 3:** Modalities of collaboration with different specialists.

	**n (%)**
**SHARED AMBULATORY**	
*Rheumatologist*	127 (51.0)
*Dermatologist*	79 (28.5)
*Ophthalmologist*	17 (6.8)
*Pneumologist*	9 (3.6)
*Endocrinologist*	7 (2.8)
*Neurologist*	5 (2.0)
*None*	91 (36.5)
**RESERVED SLOTS**	
*Rheumatologist*	180 (72.3)
*Dermatologist*	145 (58.2)
*Ophthalmologist*	56 (22.5)
*Pneumologist*	32 (12.8)
*Endocrinologist*	33 (13.2)
*Neurologist*	26 (10.4)
*None*	50 (20.1)
**ON-DEMAND VISIT**	
*Rheumatologist*	209 (83.9)
*Dermatologist*	188 (75.5)
*Pneumologist*	112 (44.9)
*Ophthalmologist*	145 (58.2)
*Endocrinologist*	111 (44.6)
*Neurologist*	107 (42.9)
*None*	6 (2.4)
**REMOTE CONSULTATION**	
*Rheumatologist*	178 (71.5)
*Ophthalmologist*	99 (39.7)
*Pneumologist*	91 (36.5)
*Endocrinologist*	89 (35.7)
*Dermatologist*	143 (57.4)
*Neurologist*	81 (32.5)
*None*	46 (18.5)

**Table 4:** Univariate analysis for factors influencing the awareness of responding physicians about IMIDs.

Outcome	Variable	OR (95% CI)	*p* Value
**Importance of IMID** (>6 on a scale from 0 to 10)			
	*Male sex*	2.37 (0.12–4.17)	0.70
	*Age > 45*	0.16 (0.2–1.55)	0.11
	*Location* *(Northern Italy)*	3.70 (0.34–33.0)	0.24
	*Specialization* *(Gastroenterology)*	Not assessable *	-
	*Type of hospital* *(Academic)*	3.36 (0.34–30.5)	0.28
	*Patient load* *(>1000 patients)*	0.43 (0.71–1.60)	0.36
**Estimated patients with concomitant IMID > 20%**			
	*Male sex*	0.76 (0.37–1.55)	0.45
	*Age > 45 years*	1.1 (0.52–2.25)	0.82
	*Location* *(Northern Italy)*	0.87 (0.43–1.77)	0.70
	*Specialization* *(Gastroenterology)*	1.36 (0.51–3.52)	0.52
	*Type of hospital* *(Academic)*	1.89 (0.90–3.9)	0.09
	*Patient load* *(>1000 patients)*	3.40 (1.42–8.12)	0.005
**Evaluation of other IMID at each visit**			
	*Male sex*	0.80 (0.35–1.66)	0.51
	*Age > 45 years*	1.05 (0.48–2.29)	0.89
	*Location* *(Northern Italy)*	1.4 (0.65–3.12)	0.37
	*Specialization* *(Gastroenterology)*	0.72 (0.20–2.55)	0.60
	*Type of hospital* *(Academic)*	2.48 (1.12–5.81)	0.03
	*Patient load* *(>1000 patients)*	1.39 (0.62–3.10)	0.40

* OR not calculable due to insufficient data in one of the groups.

## Data Availability

Data are available upon reasonable request.

## Supplementary Materials

The following supporting information can be downloaded at: https://www.mdpi.com/article/10.3390/jcm13071857/s1, The web-based 20-items questionnaire sent to Italian physicians referring to IG-IBD.
