# Supplementary material for: Immune-Mediated Inflammatory Diseases Awareness and Management among Physicians Treating Patients with Inflammatory Bowel Disease: An IG-IBD Survey
Source: J Clin Med. 2024 Mar 23;13(7):1857. doi: 10.3390/jcm13071857 (PMC11012957; doi:10.3390/jcm13071857)
Supplement: Supplementary file 1 [file jcm-13-01857-s001.zip › jcm-2814235-supplementary.pdf]

The web-based 20-items questionnaire sent to Italian physicians referring to IG-IBD

1. Which is your gender?

- Male
- Female
- No answer

2. How old are you?

- 25-34 years
- 35-45 years
- > 45 years

3. Your center is located:

- Northern Italy
- Central Italy
- Southern Italy

4. Specialization in:

- Gastroenterology
- Internal Medicine
- Other (specify)

5. Type of work environment:

- Public hospital
- Public university hospital
- Private hospital
- Private university hospital
- Other______________

6. How many years have you been following patients with IBD?

- < 5 years
- 5 – 15 years
- 15 years

7. Age of patients followed (one or more answers):

- < 14 years

- 14-17 years
- ≥ 18 years

8. Number of patients followed:

- < 500
- 500-1000
- 1000-3000
- > 3000

9. In your work environment, which of these figures are present? (multiple answers allowed)

- Rheumatologist
- Dermatologist
- Ophthalmologist
- Pulmonologist
- Endocrinologist
- Neurologist
- None of the above

10. With which of these specialists have you established a shared clinic (both specialists present at the same visit) for managing IBD patients with other IMIDs? (multiple answers allowed)

- Rheumatologist
- Dermatologist
- Ophthalmologist
- Pulmonologist
- Endocrinologist
- Neurologist
- Other (specify)
- None

11. With which of these specialists do you have the possibility to have reserved slots for a visit for your IBD patients with other IMIDs? (multiple answers allowed)?

- Rheumatologist
- Dermatologist
- Ophthalmologist
- Pulmonologist
- Endocrinologist
- Neurologist
- Other (specify)
- None

12. With which of these specialists do you have the possibility to request a visit as needed for the management of IBD cases with concomitant IMIDs? (multiple answers allowed)

- Rheumatologist
- Dermatologist
- Ophthalmologist
- Pulmonologist
- Endocrinologist
- Neurologist
- Other (specify)
- None

13. With which of these specialists do you perform remote consultations (e.g., phone consultations) as needed for a patient with IBD and another concomitant IMID? (multiple answers allowed)?

- Rheumatologist
- Dermatologist
- Ophthalmologist
- Pulmonologist
- Endocrinologist
- Neurologist
- Other (specify)
- None

14. What percentage do you estimate your IBD patients are affected by other IMIDs?

- < 10%
- 10-20%
- 20-50%
- 50-70%

15. Do you ever check for signs and symptoms compatible with concomitant IMIDs with IBD?

- Only at the first assessment
- Only if the patient reports a positive medical history
- I collect medical history data at the first assessment and actively inquire if compatible symptoms have appeared at each follow-up
- I do not routinely conduct this type of assessment

16. On a scale of 1 to 10, where 1 is "not important at all" and 10 is "extremely important," how important do you consider being aware of the presence of other IMIDs in the management of IBD patients?

17. Which of these IMIDs do you systematically investigate when collecting the patient's medical history?

- I do not consider any IMID other than IBD
- Autoimmune thyroiditis
- Type 1 diabetes mellitus
- Rheumatoid arthritis
- Ankylosing spondylitis
- Other forms of arthritis (juvenile idiopathic arthritis, psoriatic arthritis, etc.)
- Systemic lupus erythematosus
- Psoriasis
- Atopic dermatitis
- Pyoderma gangrenosum
- Erythema nodosum
- Hidradenitis suppurativa
- Ocular conditions (uveitis, iridocyclitis)
- Inflammatory lung diseases (bronchiolitis obliterans, idiopathic pulmonary fibrosis)
- Multiple sclerosis
- Primary sclerosing cholangitis
- Other liver diseases (Primary Biliary Cholangitis, autoimmune liver diseases, etc.)
- Autoimmune pancreatitis

18. How do you think IMIDs can influence the management of IBD (multiple answers possible)?

- I do not think they have any influence on the course of IBD
- Other IMIDs can negatively influence the course of IBD
- Other IMIDs can positively influence the course of IBD
- It might be necessary to make different therapeutic choices to "cover" all immune-mediated pathologies
- Some therapies indicated for one of the IMIDs might be contraindicated in IBD or *vice versa*

19. In clinical practice, in an IBD patient with concomitant IMIDs, how do you generally behave?

- The gastroenterologist independently manages all IMIDs

- The gastroenterologist takes care of IBD and delegates the management of other IMIDs to the referring specialist
- There is a multidisciplinary group that collectively manage patients with IBD and another IMID

20. Which of these specialists do you think is essential in the management of IBD with concomitant IMIDs? (only one answer)

- Rheumatologist
- Dermatologist
- Ophthalmologist
- Pulmonologist
- Endocrinologist
- Neurologist
- Other (specify)
- None
